# FOXO3a acts to suppress DNA double‐strand break‐induced mutations

**DOI:** 10.1111/acel.13184

**Published:** 2020-07-28

**Authors:** Ryan R. White, Alexander Y. Maslov, Moonsook Lee, Samantha E. Wilner, Matthew Levy, Jan Vijg

**Affiliations:** ^1^ Department of Genetics Albert Einstein College of Medicine Bronx New York USA; ^2^ Department of Biochemistry Albert Einstein College of Medicine Bronx New York USA; ^3^ Center for Single‐Cell Omics in Aging and Disease School of Public Health Shanghai Jiao Tong University School of Medicine Shanghai China; ^4^ Present address: Chemistry Department Ursinus College Collegeville Pennsylvania USA; ^5^ Present address: Vitrisa Therapeutics Durham North Carolina USA

**Keywords:** aging, bleomycin, DNA damage, DSB repair, FOXO3a, mutations

## Abstract

Genomic instability is one of the hallmarks of aging, and both DNA damage and mutations have been found to accumulate with age in different species. Certain gene families, such as sirtuins and the FoxO family of transcription factors, have been shown to play a role in lifespan extension. However, the mechanism(s) underlying the increased longevity associated with these genes remains largely unknown and may involve the regulation of responses to cellular stressors, such as DNA damage. Here, we report that FOXO3a reduces genomic instability in cultured mouse embryonic fibroblasts (MEFs) treated with agents that induce DNA double‐strand breaks (DSBs), that is, clastogens. We show that DSB treatment of both primary human and mouse fibroblasts upregulates FOXO3a expression. FOXO3a ablation in MEFs harboring the mutational reporter gene lacZ resulted in an increase in genome rearrangements after bleomycin treatment; conversely, overexpression of human FOXO3a was found to suppress mutation accumulation in response to bleomycin. We also show that overexpression of FOXO3a in human primary fibroblasts decreases DSB‐induced γH2AX foci. Knocking out FOXO3a in mES cells increased the frequency of homologous recombination and non‐homologous end‐joining events. These results provide the first direct evidence that FOXO3a plays a role in suppressing genome instability, possibly by suppressing genome rearrangements.

## Graphical abstract

Gene families associated with conserved pathways of aging, such as sirtuins and FoxOs, have been shown to act to enhance DNA repair. We show that DNA double‐strand breaks upregulate expression of FOXO3A in cells and that FOXO3A is capable of suppressing mutagenic genome rearrangements.

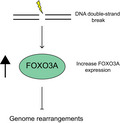

## 1 INTRODUCTION, RESULTS, AND DISCUSSION

Extensive evidence supports the notion that somatic genome alterations are fundamental to aging, not only giving rise to cancer but possibly also causing non‐cancer, age‐related degeneration and disease (Kennedy, Loeb, & Herr, 2012; Vijg & Suh, 2013). Indeed, one defining characteristic of aging is the accumulation of somatic mutations and DNA damaging lesions arising from endogenous or environmental agents (Dolle et al., 1997; Martincorena et al., 2015; Maslov et al., 2013). Moreover, we have recently shown that DNA double‐strand breaks (DSBs) are capable of accelerating multiple aging pathologies in otherwise normal, young mice (White et al., 2015).

Certain gene families, such as sirtuins and FoxOs, have been linked to longevity in model organisms by regulating multiple signaling pathways in response to stress, including DNA damage (Guarente, 2011; van der Horst & Burgering, 2007). Genetic variants found in FOXO3a have been associated with extreme human longevity in multiple ethnic backgrounds (Anselmi et al., 2009; Broer et al., 2015; Flachsbart et al., 2009; Willcox et al., 2008). *Foxo3a*‐deficient mice are viable, yet females display reduced ovarian follicle activation, a feature of premature ovarian aging (Castrillon, Miao, Kollipara, Horner, & DePinho, 2003; Hosaka et al., 2004). FOXO3a has also been shown to stimulate DNA repair in response to oxidative stress (Tran et al., 2002), while other studies suggested FOXO3a activates p53 to initiate a pro‐apoptotic program in response to DNA damage (Chung et al., 2012). Together, these studies provide evidence for FOXO3a playing a role in promoting tissue homeostasis in response to stress, offering a possible explanation for its role in lifespan extension. Hence, we reasoned that involvement of FOXO3a in stress response and longevity could rely in part on its ability to maintain genome stability (Charitou & Burgering, 2013). Here, we directly assessed the role FOXO3a plays in maintaining genome stability in response to DSBs.

**FIGURE 1:**
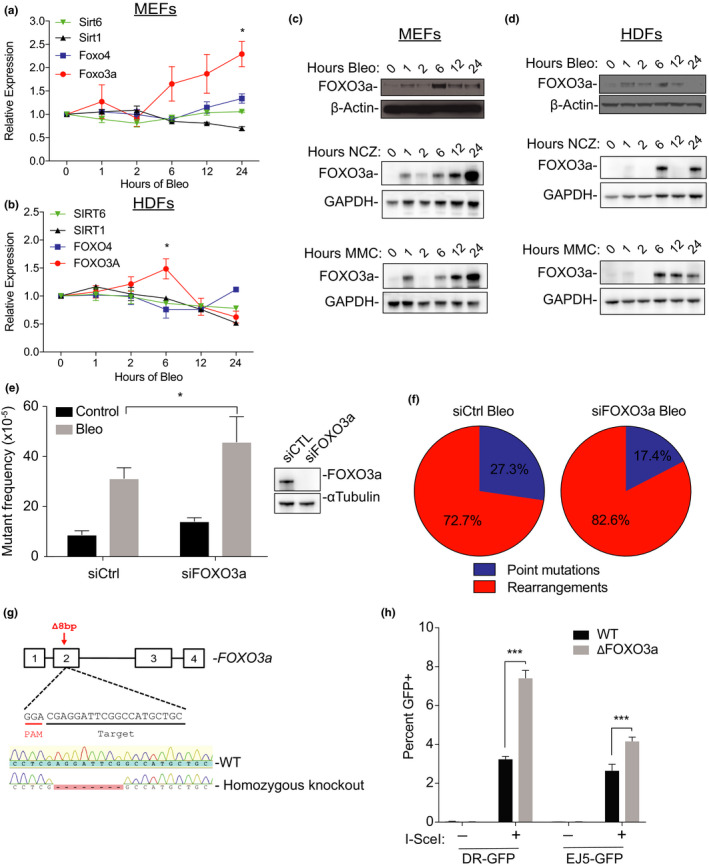
FOXO3a responds to DNA double‐strand breaks and mitigates genome rearrangements. (a) MEFs were treated with 1.4 μM of bleomycin (Bleo) for up to 24 hr. Expression of SIRT1, SIRT6, FOXO3a, and FOXO4 was analyzed in triplicates by qPCR normalized to 18s rRNA. (b) HDFs were treated with 2.8 μM of bleomycin for up to 24 hr. Expression of SIRT1, SIRT6, FOXO3a, and FOXO4 was analyzed in triplicates using qPCR and normalized to 18s rRNA. Shown are the mean values ± *SD*. where *n* = 3. *p*‐Values were calculated using Student's *t* test. *p < 0.05 (c,d) Western blot of FOXO3a in whole‐cell lysates from MEFs (c) and HDFs (d) after treatment with 1.4 μM bleomycin, 0.5 mg/ml Neocarzinostatin (NCZ) and 50 nM mitomycin C (MMC) for the indicated times. (e) lacZ mutant frequency from the rescue assay of either siCtrl or siFOXO3a MEFs treated with or without (control) 1.4 μM bleomycin for 3 days. Values represent the mean mutation frequency ± *SD* where *n* = 3. Western blot shows levels of FOXO3a knockdown at 48 hr. (f) Spectrum of mutant lacZ plasmids rescued from siCtrl or siFOXO3a MEFs with bleomycin. Values are given as the percentage of point mutations or genome rearrangements out of the total number of mutants screened, where *n* = 48 mutant colonies screened from each of the biological triplicates. (g) Generation of a FOXO3a knockout clone in mouse ES cells using CRISPR‐Cas9 with a single guide RNA targeting the second exon. The selected KO clone had a homozygous 8 bp deletion causing a frameshift and a premature stop codon, confirmed by Sanger sequencing. Wild‐type (WT) and FOXO3a KO cells were then targeted with either DR‐GFP or EJ5‐GFP constructs. (h) Reporter lines were then transfected with I‐SceI or control vector and allowed to recover for 3 days before scoring GFP+ repair events using flow cytometry. Experiments were performed in triplicate and >20,000 cells analyzed per sample. Values represent the mean percentage of GFP recombinants out of the total number of the parental population ± *SD*. *p*‐values were calculated using Student's *t* test. ***p < 0.005

We first tested if expression of FOXO3a and other FoxOs and sirtuins found associated with longevity was upregulated at the transcript level in response to DNA damage. Using bleomycin, a potent inducer of DNA DSBs, primary mouse embryonic fibroblasts (MEFs) were treated for up to 24 hr. Our results show that FOXO3a mRNA is upregulated from about 6 hr until at least 24 hr post‐treatment; at that time, it was upregulated ~2.2‐fold as compared with untreated, control cells (Figure 1a, *p* < 0.05), while SIRT1, SIRT6, and FOXO4 remained relatively unchanged. To confirm this response was not specific for mouse cells, we also tested this regimen with primary human dermal fibroblasts (HDFs). Again, FOXO3a mRNA levels increased, but only up until ~1.5‐fold at 6 hr post‐treatment, after which it decreased again (Figure 1b). We also observed upregulation of FOXO3a, in response to bleomycin and compared to untreated MEFs, in both MEFs and HDFs, at the translational level. In this case, we also analyzed the response to other mutagens, that is, neocarzinostatin (NCZ), and mitomycin C (MMC), which was similar to the response to bleomycin (Figure 1c,d). Together, these results show that FOXO3a is upregulated at both the transcriptional and translational levels in response to DNA damage.

Since FOXO3a increases in response to DNA damage, we wanted to ascertain the role that FOXO3a might play in the mutational outcome arising from DSBs. To do this, we took advantage of a lacZ mutational reporter system previously described by our laboratory (Boerrigter, Dolle, Martus, Gossen, & Vijg, 1995). Using siRNA, we depleted FOXO3a in MEFs harboring the lacZ reporter (Figure 1e), then treated them with bleomycin 48 hr after siRNA transfection and harvested the cells at 3 days post‐treatment, allowing time for the damage to be repaired and mutations to become fixed, as we have shown previously (Quispe‐Tintaya et al., 2016, 2018). Once MEFs were harvested, the lacZ‐containing plasmid was recovered from its integrated state in the genome and transferred into *E. coli* to select for mutations (Garcia et al., 2007). Knockdown of FOXO3a in bleomycin‐treated MEFs resulted in an increased mutant frequency as compared to control siRNA cells (45.9 × 10^–5^ vs. 31.3 × 10^–5^; Figure 1e). We next characterized the spectra of the mutant lacZ plasmids rescued from the MEFs. Of note, mutants showing no size‐change after restriction digestion are considered to be point mutations, while those that do show a size‐change are considered genome rearrangements (Garcia et al., 2007). All excessive mutations in bleomycin‐treated MEFs after FOXO3a knockdown were genome rearrangements, as evidenced by ~10% increase of this type of mutation as compared to treated control MEFs (82.6% vs. 72.6%, respectively; Figure 1f). We also performed cellular sensitivity assays to MMC and NCZ in FOXO3a‐depleted cells, showing that these cells are not overly sensitive to these damaging agents (Figure S1a,b). Together, these results indicate that FOXO3a deficiency confers susceptibility to mutation accumulation but not cell death, specifically genome rearrangements arising from DSBs.

**FIGURE 2:**
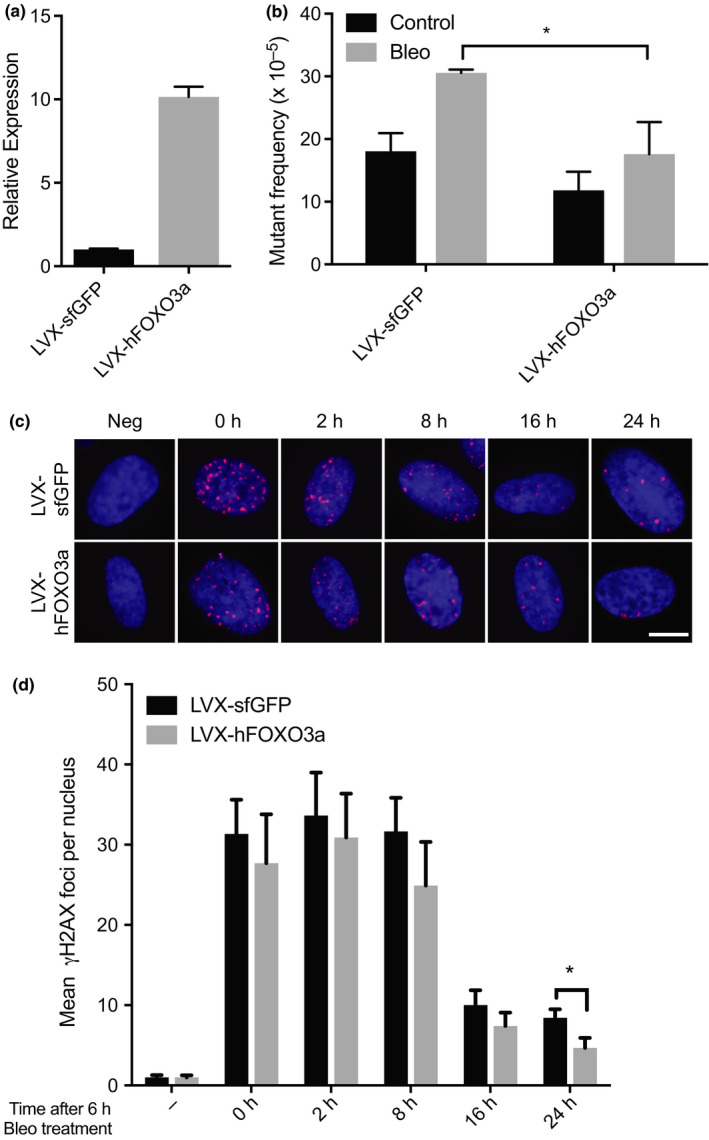
FOXO3a overexpression suppresses bleomycin‐induced mutations and increases clearance of DNA damage foci. (a) MEFs were transduced with LVX‐sfGFP or LVX‐hFOXO3a. Confirmation of hFOXO3a overexpression was performed using qPCR. Levels of hFOXO3a overexpression were compared with endogenous mouse FOXO3a in the LVX‐sfGFP MEFs and normalized to mouse Gapdh. (b) lacZ mutant frequency from the rescue assay of either LVX‐sfGFP or LVX‐hFOXO3a transduced MEFs treated 1.4 μM bleomycin for 3 days. Values represent the mean mutation frequency ± *SD* where *n* = 3. (c‐d) HDFs expressing LVX‐sfGFP or LVX‐hFOXO3a were treated with 2.8 μM of bleomycin for 6 hr. Cells were then washed with PBS and medium replaced. HDFs were fixed at the indicated times post‐bleomycin treatment and stained for γH2AX damage foci. (c) Representative images. Scale bar = 10 μm (d) Quantification of γH2AX foci per nucleus. Values represent the mean ± *SD* of foci per nucleus, where >100 nuclei were scored per time point per sample. *p*‐values were calculated using Mann–Whitney test. *p < 0.05

Since FOXO3a deficiency can cause an increase in mutation accumulation, we next asked whether increased FOXO3a could suppress mutations. To address this, we overexpressed human FOXO3A via lentiviral‐mediated transduction into lacZ MEFs (hFOXO3a). hFOXO3a was overexpressed in MEFs ~10‐fold (Figure 2a) as compared to endogenous FOXO3a as assayed by qPCR; control MEFs only expressed sfGFP. MEFs expressing either sfGFP or FOXO3A were treated with bleomycin and allowed to recover for 3 days. The results showed that bleomycin treated hFOXO3a expressing MEFs had a significantly lower mutant frequency than control sfGFP MEFs (17.6 × 10^–5^ vs. 30.4 × 10^–5^; *p* < 0.05, Figure 2b). Therefore, FOXO3a overexpression acts to suppress bleomycin‐induced genome instability, possibly by reducing erroneous repair of DSBs.

Considering overexpression of FOXO3a is capable of suppressing mutations arising from DSBs induced by bleomycin, we next examined the effect of FOXO3a overexpression on DSB repair foci resolution. Here, we treated HDFs, expressing either sfGFP or hFOXO3a, with bleomycin for 6 hr and analyzed γH2AX foci at 0, 2, 8, 16, and 24 hr after treatment (Figure 2c,d). There were noticeably less γH2AX foci in cells overexpressing FOXO3A, a situation that persisted 24 hr after treatment, where on average there were about half the number of γH2AX foci in LVX‐FOXO3a cells as compared to control LVX‐sfGFP HDFs (*p* < 0.05; Figure 2d). These results show that overexpression of FOXO3a hinders DNA damage foci appearance by potentially accelerating their clearance.

Previous studies have shown activation of FoxO transcription factors can antagonize the cell cycle in a cyclin D1‐dependent manner in immortalized cell lines (Kops et al., 1999; Schmidt et al., 2002). Thus, to determine whether mutation accumulation and foci clearance are cell cycle specific, we analyzed the cell cycle progression in response to alterations in FOXO3a expression. In primary MEFs or HDFs where FOXO3a was depleted by siRNA, we did not observe any differences in the phases of the cell cycle (Figure S1d,f). However, when we analyzed primary MEFs overexpressing hFOXO3a, we did observe a slight decrease in the S‐phase population, 19.9% versus 25.3% in control sfGFP expressing cells, which was accounted for by an increase in G_0/1_ phase (Figure S1e). Considering these findings, FOXO3a may have a slight, species‐specific effect on cell cycle regulation, in the context of overexpression, but does not overall drastically alter cell cycle progression.

Considering deficiency of FOXO3a gives rise to an increase in genome rearrangements in response to DSB‐induction (Figure 2c), we next asked whether FOXO3a functions in a specific DSB repair pathway, that is, homologous recombination (HR) or non‐homologous end joining (NHEJ). To test this, we utilized two reporter systems, the DR‐GFP reporter to assay HR and the EJ5‐GFP reporter to assay NHEJ (Bennardo, Cheng, Huang, & Stark, 2008; Kass et al., 2013). First, we knocked out FOXO3a in mouse ES cells by using clustered regularly interspaced short palindromic repeats (CRISPR)‐Cas9‐mediated genome editing targeted to exon 2 (Figure 1g) (Cong et al., 2013). Using this approach, we isolated a clone with a homozygous 8 bp deletion in FOXO3a, confirmed by Sanger sequencing (Figure 1g). Next, we targeted both DR‐GFP and EJ5‐GFP reporter systems into wild‐type and ΔFOXO3a cells and screened for positive integration of both. Wild‐type and ΔFOXO3a cells containing either DR‐GFP or EJ5‐GFP reporters were then transiently transfected with I‐SceI and allowed to recover for 3 days before assessed by flow cytometry for GFP+ cells. We found that ΔFOXO3a cells had a twofold increase in GFP+ recombinants in the HR reporter assay, 3.5% versus 7.4% (Figure 1h). In the cells harboring the NHEJ reporter, we also observed a significant increase in the ΔFOXO3a cells from 2.7% to 4.2% (Figure 1h). These results suggest FOXO3a acts to restrain both of these DSB repair pathways, possibly suppressing mutagenic repair after a DSB is detected but before a repair pathway choice is made.

Taken together, our data indicate that FOXO3a is capable of modulating DNA double‐strand break repair, possibly making it less error prone, to maintain genome stability. This finding is consistent with previous evidence that suggest FOXO3a mediates the stress response to genomic damage (Brunet et al., 2004; Tran et al., 2002). Specifically, our data show that FOXO3a (a) is upregulated in response to clastogenic agents, (b) acts as regulator of genome maintenance by suppressing mutations, namely genome rearrangements, by potentially accelerating DNA damage foci clearance. Our studies offer new insight into a previously unknown role for FOXO3a in promoting DNA repair in response to genomic damage. Additional work to understand how FOXO3A directly interacts with the DSB repair machinery may uncover a novel mechanism to maintain tissue homeostasis in response to genomic stress, ultimately promoting cellular and organismal longevity.

## CONFLICT OF INTEREST

JV is a founder of Singulomics Corp.

## AUTHOR CONTRIBUTIONS

R.R.W. and J.V. designed the experiments, analyzed the data, and wrote the manuscript. R.R.W., A.Y.M., S.E.W., M.L., and M.L performed the experiments. All authors approved the final version of the manuscript.

## Supporting information

Fig S1

Supplementary Material
