# Supplementary material for: FOXO3a acts to suppress DNA double‐strand break‐induced mutations
Source: Aging Cell. 2020 Jul 28;19(9):e13184. doi: 10.1111/acel.13184 (PMC7511859; doi:10.1111/acel.13184)
Supplement: Supplementary file 2 — Supplementary Material [file ACEL-19-e13184-s002.docx]

**Supporting Information**

**Experimental Procedures**

**Animals and tissue collection.** All procedures involving animals were approved by the Institutional Animal Care and Use Committee (IACUC) of Albert Einstein College of Medicine. C57Bl/6 LacZ line 30 mice used in this study were described previously (Boerrigter *et al.* 1995).

**Cell culture.** Primary mouse embryonic fibroblasts were isolated from pregnant LacZ mice at day E13.5, according to routine procedure. Each line was derived from a single embryo. MEFs and primary HDFs were cultured in complete fibroblast medium (DMEM, 15% fetal bovine serum, non-essential amino acids, 1X GlutaMax, sodium pyruvate, and 100 units/ml penicillin-streptomycin, in a 37 °C incubator at 3% O_2_ and 10% CO_2_. Bleomycin sulfate (MilliporeSigma) was dissolved in 1X PBS and used for MEFs at a final concentration of 2 μg/mL and for HDFs at 4 μg/mL for treatments unless stated otherwise. Where noted, bleomycin was washed off of fibroblasts 6 hours after addition to medium using 1X PBS. Neocarzinostatin (MilliporeSigma) was added to cells as indicated for 30 minutes then washed off twice with 1X PBS before adding complete media back onto cells. Mitomycin C (MilliporeSigma) was dissolved in sterile water and added to cells at the indicated doses in complete medium with no wash off. DR-GFP and EJ5-GFP constructs were targeted to the *Pim1* locus of the ES cells as previously described (Bennardo *et al.* 2008; Kass *et al.* 2013). ES cells were cultured in feeder-layer free conditions in complete ES medium, as previously described (Richardson *et al.* 1998), in a 37 °C incubator at 10% CO_2_.

**siRNA.** MEFs (2 x 10^6^) were transfected with 20 μM of either ON-TARGETplus Non-targeting Pool or ON-TARGETplus Mouse Foxo3a SMARTpool (040728) siRNA (Dharmacon), using the Amaxa Nucleofector program A-024 with the MEF1 kit (Lonza). MEFs were then resuspended in complete medium and allowed to recover for 48 hours before plating for subsequent experiments.

**Lentivirus**. Low passage HEK293T cells were plated to 50% confluency into a 60mm dish. pLVX-sfGFP or pLVX-FOXO3a was mixed with 2^nd^ generation lentiviral packing plasmids (psPAX2 and pMD2.G) and transfected into HEK293T cells using XtremeGene 9 DNA transfection Reagent (Roche). Virus containing media was filtered through a 0.45 μM low-protein binding filter (Milipore) and added to MEFs or HDFs each day for 3 consecutive days post transfection. Transduction efficiency was scored by analyzing for GFP+ cells and generally >90% efficiency was achieved with each viral transduction.

**qPCR.** Total RNA was isolated from cells or tissues using miRNeasy Mini kit according to the manufacturers protocol (Qiagen). RNA was converted into cDNA using SuperScript IV First-strand Synthesis kit (Invitrogen) using either random hexamers or oligo dT. qPCR was performed using 50 ng of cDNA using ABI StepOne Plus system for TaqMan^®^ system (Applied Biosystems, Inc.). All calculations were performed using the ΔΔCT method with TaqMan assays normalized to 18s rRNA or GAPDH where indicated with all biological replicate values representing the mean of technical triplicates. Taqman assay ID’s used: Foxo3-Mm01185722_m1, Foxo4-Mm00840140_g1, Sirt6-Mm01149042_m1, Sirt1-Mm00490758_m1, Rn18s-Mm03928990_g1, Gapdh-4352661, GAPDH-Hs02758991_g1, FOXO3-Hs00921424_m1, FOXO4-Hs00936217_g1, SIRT6-Hs00213036_m1, SIRT1-Hs01009006_m1 (Applied Biosystems, Inc.).

**LacZ mutation assay.** Mutation frequencies at the integrated lacZ reporter were determined as described (Dolle *et al.* 1997; Garcia *et al.* 2007). Briefly, genomic DNA was digested with HindIII and the lacZ containing fragments isolated using magnetic beads precoated with lacZ/lacI fusion protein. After washing, DNA was eluted from the beads using isopropylthio-β-galactoside (IPTG), circularized with T4 ligase and transformed into electrocompetent E. coli C (∆lacZ, galE-). To determine the total number of transformants, 0.1% of the transformed bacterial cells were plated in agar containing X-gal. The remainder was plated in 0.3% P-gal to select for cells harboring plasmids with lacZ mutations. Mutation frequencies were calculated as the number of mutant colonies divided by the number of recovered transformants.

**Cellular sensitivity assay.** HPV E6/E7-transformed BJ fibroblasts were plated into six-well plates at 3.5x10^4^ cells per well in triplicate 24 hours after transfection with siRNA. 24 hours after plating, cells were treated with the indicated drugs at the indicated dose in complete medium. Mitomycin C was left on for 4 days splitting cells and replacing with complete medium and allowing cells to recover for 2-3 days. Neocarzinostatin was left on cells for 30 minutes and then cells washed twice with PBS and replacing with complete medium. Cells were harvested and counted on day 7-8 post treatment and cell numbers normalized to an untreated well of cells to give percent survival.

**Cell cycle analysis.** MEFs or HDFs were plated in fibroblast medium at 1 x 10^6^ cells per 100mm dish and allowed to recover and adhere for 24 hours. HDFs were pulse labeled with 10 μM of EdU for 2 hours in fibroblast medium and detected using the Click-iT EdU Flow Cytometry Assay Kit (Life Technologies) according to the manufacturers protocol. HDFs were counterstained with FxCycle Violet (Life Technologies) stain for DNA content for 30 min at room temperature prior to analysis. MEFs and HDFs were analyzed using flow cytometry on a DxP10 FACSCalibur (Becton Dickinson) where 100,000 events were analyzed per sample. Flow cytometry data was analyzed using FlowJo version 10.0.8r1.

**Immunofluorescence and microscopy**. HDFs were fixed for 15 min at room temperature using 4% formaldehyde. Staining was carried out using standard protocols. Primary antibodies were anti-Phospho-Histone H2A.X (Ser139) at 1:1000 (Cell Signaling). Secondary antibody was AlexaFluor 594 donkey anti-rabbit (Invitrogen) at 1:1000. Slides were mounted with ProLong Diamond Antifade reagent with DAPI (Invitrogen) and allowed to cure overnight at room temperature. Images were acquired on a Zeiss Axio Imager M2 using a 63X oil objective. Images were analyzed blindly using Volocity software where background thresholds for foci were set using control untreated samples.

**CRISPR targeting.** ES cells were plated onto gelatinized plates and co-transduced with pCM57.1-Flag-CAS9 and pLX304-VB032sgRNA lentivirus at an MOI 1 with polybrene at 8 μg/ml. Cells were then selected in puromycin at 10 μg/ml and blasticidin at 5 μg/ml for 10 days. Selected ES cells (1 x 10^5^) were then plated onto a 100mm in the presence of doxycycline at 1 μg/ml for 8 days. Clones were then manually picked and expanded for Foxo3a deletion screening by Sanger sequencing and western blotting.

**DSB repair assay.** Targeted DR-GFP, DR-GFP (ΔFoxo3a), EJ5-GFP, and EJ5-GFP (ΔFoxo3a) mouse ES cells were plated at 0.38 x 10^4^ cells per cm^2^ plate in complete ES medium. The next day ES cells were transfected with 1.0 μg of pCBASce or empty vector mixed with 1.5 μL of Lipofectamine 3000 reagent and 2 μL of P3000 reagent in serum-free DMEM. Media was replaced with complete ES medium 12 hours after transfection. ES cells were analyzed via flow cytometry on a FACSCanto II (Becton Dickinson) for GFP+ cells three days after transfection, where at least 20,000 events were analyzed per sample. Flow cytometry data was analyzed using FlowJo version 10.0.8r1.

**Figure S1. Cell cycle analysis and expression of FOXO3a in aged tissues.** (A, B) Cellular sensitivity assays in E6/E7-transformed BJ fibroblasts depleted of FOXO3a by siRNA to (A) Mitomycin C, MMC and (B) Neocarzinostatin, NCZ. Error bars represent standard deviation, n= 3. (C) Western blot of FOXO3a after knockdown by siRNA in BJ cells for sensitivities in A and B. (D-F) Cell cycle was assessed after pulse labeling in the indicated cell types and perturbations with EdU for 1 hour followed by staining with Click reaction and DNA content stained by FxViolet. Cells were then analyzed by flow cytometry and percent values of whole were plotted. Mean value is shown in red and error bars represent standard deviation, n=3.

**References**

Bennardo N, Cheng A, Huang N, Stark JM (2008). Alternative-NHEJ is a mechanistically distinct pathway of mammalian chromosome break repair. *PLoS genetics*. **4**, e1000110.

Boerrigter ME, Dolle ME, Martus HJ, Gossen JA, Vijg J (1995). Plasmid-based transgenic mouse model for studying in vivo mutations. *Nature*. **377**, 657-659.

Dolle ME, Giese H, Hopkins CL, Martus HJ, Hausdorff JM, Vijg J (1997). Rapid accumulation of genome rearrangements in liver but not in brain of old mice. *Nature genetics*. **17**, 431-434.

Garcia AM, Busuttil RA, Rodriguez A, Cabrera C, Lundell M, Dolle ME, Vijg J (2007). Detection and analysis of somatic mutations at a lacZ reporter locus in higher organisms: application to Mus musculus and Drosophila melanogaster. *Methods Mol Biol*. **371**, 267-287.

Kass EM, Helgadottir HR, Chen CC, Barbera M, Wang R, Westermark UK, Ludwig T, Moynahan ME, Jasin M (2013). Double-strand break repair by homologous recombination in primary mouse somatic cells requires BRCA1 but not the ATM kinase. *Proc Natl Acad Sci U S A*. **110**, 5564-5569.

Richardson C, Moynahan ME, Jasin M (1998). Double-strand break repair by interchromosomal recombination: suppression of chromosomal translocations. *Genes & development*. **12**, 3831-3842.
